# Exploring Emotion Regulation and Perceived Control as Antecedents of Anxiety and Its Consequences During Covid-19 Full Remote Learning

**DOI:** 10.3389/fpsyg.2021.675910

**Published:** 2021-07-01

**Authors:** Ting Zhao, Zongmei Fu, Xi Lian, Linning Ye, Wei Huang

**Affiliations:** ^1^School of Foreign Languages for Business, Southwestern University of Finance and Economics, Chengdu, China; ^2^School of English Education, Guangdong University of Foreign Studies, Guangzhou, China; ^3^Department of Computer Science, Southwestern University of Finance and Economics, Chengdu, China

**Keywords:** cognitive reappraisal, expressive suppression, perceived control, anxiety, Covid-19 remote learning

## Abstract

Maintaining the emotional well-being of learners during a pandemic is important. This study explored the effects of two emotion regulation strategies (cognitive reappraisal, expressive suppression) and perceived control on full remote learners' anxiety during Covid-19, and their relationship to perceived learning. Structural equation modeling was used to analyze 239 questionnaires completed by Chinese graduate students taking a course remotely from home for 13 weeks. This study showed that reappraisal was positively related to perceived control, whereas suppression was negatively related to perceived control. Reappraisers perceived more learning, whereas suppressors experienced more anxiety. Anxiety was significantly and negatively related to perceived learning. Mediation analyses showed the existence of different patterns of mediation in the pathways from the two types of emotion regulation to perceived learning. These findings are discussed in relation to relevant studies conducted during non-pandemic periods and Covid-19, and based on the results we highlight the need for interventions aimed at developing adaptive emotion regulation strategies and reducing anxiety in emergency remote learning.

## Introduction

On March 11, 2020, the World Health Organization (WHO) announced Covid-19 (coronavirus disease) as a pandemic (WHO Regional Office for Europe, 2020). Learning in brick-and-mortar classrooms was urgently suspended and replaced by full remote learning. This sudden change placed a severe psychological burden on learners (Pakpour et al., 2020; Sycamore, 2020), in addition to the negative emotions directly or indirectly caused by the pandemic, such as anxiety about being infected, loneliness due to lockdowns, and worries over financial strain and academic delays (AlAteeq et al., 2020; Cao et al., 2020; Gubler et al., 2020; Husky et al., 2020; Savitsky et al., 2020).

Emotions are critical to learning (Zull, 2006). Evidence across multiple disciplines, including psychology, education, and neuroscience, consistently shows that the frequency and intensity of academic emotions can contribute to or interfere with learning processes and academic achievement (Pekrun et al., 2011; Seli et al., 2016; Tyng et al., 2017). Anxiety exacerbated during Covid-19 remote learning is thought to be a barrier to learning success (Gillis and Krull, 2020). It is important to understand how learners' anxiety in such emergency remote learning (ERL) situations arises and the mechanisms that trigger this negative emotion.

Perceived control has been shown to be an important antecedent of anxiety in learning (e.g., Goetz et al., 2006; Marchand and Gutierrez, 2012; Shao et al., 2020). Students' perceived control over Covid-19 remote learning was found to vary from individual to individual (Dhawan, 2020). The use of specific emotion regulation strategies has also been associated with different levels of anxiety (Gross and John, 2003; Hofmann et al., 2009; Schutte et al., 2009; but also see Eastabrook et al., 2013). These strategies can be broadly categorized into *antecedent-focused* (e.g., cognitive reappraisal) and *response-focused* strategies (e.g., suppressing emotions) (Gross and John, 2003; Gross, 2013). Over the years, different theoretical models have emphasized the classification of these strategies as adaptive or maladaptive (Aldao et al., 2010). Two strategies of regulation that have long been considered adaptive and maladaptive are reappraisal and suppression, respectively (Aldao et al., 2010).

The present study primarily aimed to test a model of proposed relationships between emotion regulation (cognitive reappraisal, expressive suppression), perceived control, anxiety as an achievement emotion, and perceived learning during Covid-19 remote learning. Perceived learning is indicative of student achievement (Rockinson-Szapkiw et al., 2016). The present study also aimed to explore the mediation effects of perceived control and anxiety on the association between the two types of emotion regulation and perceived learning. The results of this study will hopefully advance our understanding of anxiety associated with ERL during global public health crises and the mechanisms by which it acts on learning outcomes, and in practice, provide evidence-based recommendations on how learning-related anxiety can be effectively reduced in ERL.

## Literature Review and Hypothesis Development

### Anxiety and Covid-19 Remote Learning

Emotions in academic contexts were once neglected because they were considered contrary to rational thinking and hindered effective teaching and learning (Cleveland-Innes and Campbell, 2012). However, emotions cannot be ignored, as learning has been found to be fraught with emotional experiences (Dirkx, 2008). A series of studies by Pekrun et al. (2002, 2004, 2011) have shown that learners often experience a variety of, both positive and negative, emotions during traditional learning. Research on online learning has also shown that the emotions felt by learners vary (e.g., Artino and Jones, 2012; Tempelaar et al., 2012).

According to models that categorize emotions (Schlosberg, 1954; Russell, 1980), emotions can be perceived as varying along *valence* (positive-negative) and *arousal* (low-high intensity). In terms of *object focus* (Pekrun, 2006), achievement emotions can be distinguished into activity emotions (e.g., in studying situations) and outcome emotions (e.g., facing success and failure). According to valence, arousal, and object focus, anxiety is commonly described as a negatively valenced emotion with high intensity, and it can be elicited during an activity or by an outcome. Anxiety is produced by a combination of multiple components involving uneasiness, nervousness, worries, avoidance motivation, and related peripheral physiological activity (Pekrun, 2006). Anxiety, among all emotions, is one of the most frequently studied in technology-based learning environments (Loderer et al., 2020). In the present study, we chose to focus on anxiety, in part because it has a strong research base and great value for attention, especially during a global pandemic outbreak.

Emotions that arise in online learning are related to self, others, task, and technology (Wosnitza and Volet, 2005). Evidence suggests that online students' anxiety may be due to the following aspects: unpreparedness to learn an online course (Abdous, 2019), working with “unknown others” (Hilliard et al., 2020), course-specific demands and requirements (Zembylas, 2008), and unfamiliarity with information technology (Fuller et al., 2016). During Covid-19, learners experienced some anxieties specific to emergency periods. Changes in ways of learning and learning environments are a contributing factor to anxiety. These changes are sudden and compulsive in nature (Bozkurt and Sharma, 2020). The transition to ERL results in the absence of a scheduled university environment, and a shift to a home environment, which poses a significant challenge for many students (Sycamore, 2020). With learning and classroom routines disrupted, students struggled with the transition to remote learning (Biber et al., 2020). In addition, remote learners' anxiety during Covid-19 has been found to be associated with the following aspects: falling academically behind other peers (Pakpour et al., 2020), increased academic workload (Wang et al., 2020c), and uncertainty about academic performance (Cao et al., 2020).

### Antecedents and Consequences of Anxiety

An important antecedent of anxiety is related to reduced perceived sense of control (Roseman and Evdokas, 2004; Pekrun, 2006). Perceived control is conceptualized as one's perceived ability to shape or influence an event, taking into account situational demands, coping potential, and regulatory ability (Lazarus and Folkman, 1984). In the field of education, perceived academic control specifically refers to the degree of control learners perceive they have over the impact of academic outcomes. Perceived control is closely related to another concept, self-efficacy, because both fall within the expectancy component of self-concept (Pintrich and De Groot, 1990). Perceived academic control is a relatively stable psychological disposition (encompassing both state-like and trait-like components) that affects learners' motivation and achievement striving (Perry et al., 2001, 2005b).

According to Pekrun's (2006) control-value theory (CVT), perceived control plays a pivotal role in influencing achievement emotions. Studies applying this theory in traditional classrooms reveal that low perceived control is associated with increased anxiety and low academic achievement (e.g., Perry et al., 2001; Pekrun et al., 2004; Goetz et al., 2006). Although few studies have applied CVT to the study of online learners, Marchand and Gutierrez (2012) found that self-efficacy was negatively related to learning anxiety. This pattern of relationship was replicated in science learning during Covid-19 (Yang X. et al., 2020).

The association between anxiety and learning outcomes has been inconsistent in the literature. Some studies observed negative associations (Pekrun et al., 2009; Artino et al., 2010; You and Kang, 2014), and even, as CVT suggests, anxiety partially mediated the relationship between control and achievement (Butz et al., 2015). Other studies, however, found non-significant associations (Tempelaar et al., 2012; Heckel and Ringeisen, 2019). These inconsistent results may be related to the different levels of anxiety in the investigated samples. Drawing on *attentional control theory* (Eysenck et al., 2007), high levels of anxiety may impair performance because of attentional control deficits in the ability to maintain task goals. However, low or moderate levels of anxiety may lead individuals to devote more resources and effort to maintaining performance. In a study conducted during Covid-19, Biber et al. (2020) analyzed the questionnaires about online learning completed by 1,640 students enrolled in a wellness course in the spring semester of 2020. Anxiety was shown to be negatively related to perceived instructional effectiveness.

### Emotion Regulation: Reappraisal and Suppression

Emotions are regulatory in nature. Emotion regulation strategies regulate (increase, maintain, or decrease) emotions by intervening in specific stages of the emotion production system using conscious and non-conscious strategies (Gross, 2001). Depending on the time point of the intervention, antecedent-focused emotion regulation occurs before full activation of emotional responses, whereas response-focused emotion regulation strategies are used following emotional activation (Gross, 1998). According to the process model of emotion regulation, antecedent-focused strategies include situation selection, situation modification, attentional deployment, and cognitive change, whereas response-focused strategy is represented by response modulation (Gross, 1998, 2002; Gross and Thompson, 2007).

The strategies representing antecedent-focused and response-focused emotion regulation families, respectively, are cognitive reappraisal (a cognitive-change strategy) and expressive suppression (a response-modulation strategy). These two strategies have been studied most frequently compared to other emotion regulation strategies. Reappraisal regulates an emotional impact by changing the way events are evaluated, and operates primarily through meaning-evaluation mechanisms (Gross, 1998, 2001). It overlaps conceptually to some extent with positive reinterpretation (Carver et al., 1989) in the coping literature. Specific methods of reappraisal are *recontrual* (i.e., changing the situational construal) and *repurposing* (i.e., changing the goal set) (Uusberg et al., 2019). An example of reappraisal is when remote learners try to see the technical difficulties encountered on a distance learning platform as an opportunity to learn new technologies. Emotion suppression involves inhibiting or hiding the emotional reactions that have arisen (Gross and John, 2003). An example of suppression is that the negative emotions triggered by the difficulties and challenges encountered by learners in Covid-19 remote learning are not expressed, but hidden.

According to the appraisal model that aims to unify appraisal, emotion regulation, and emotion generation (Yih et al., 2019), changes in appraisal are one mechanism by which emotion regulation strategies regulate emotions. One of the underlying components of appraisal is likelihood, which includes certainty about the current situation as well as future expectations and outcome probabilities. Research in psychology has shown that regulation strategies have an effect on perceived control (Fontaine et al., 1993; Dijkstra and Homan, 2016). In Dijkstra and Homan (2016), perceived sense of control was identified as an important explaining variable in the relationship between regulation and psychological health.

A large body of research has shown that reappraisal is associated with less negative affect and more well-being, whereas suppression is associated with more negative affect and less well-being (Gross and John, 2003; Hofmann et al., 2009; Schutte et al., 2009; Ehring et al., 2010; Dijkstra and Homan, 2016; Low et al., 2017; but see Yeung and Fung, 2012; Eastabrook et al., 2013, for diverging findings). Unlike suppression as a response-focused strategy, reappraisal is antecedent-focused and is typically used before emotions unfold and, therefore, consumes fewer cognitive resources (Cutuli, 2014).

Reappraisal and suppression have been studied in traditional learning and test-taking situations (e.g., Nett et al., 2011; Sorić et al., 2013; Ben-Eliyahu and Linnenbrink-Garcia, 2015). These two emotion regulation strategies in online learning have received some (albeit limited but emerging) academic attention. For example, in Webster and Hadwin (2015), online learners were found to reappraise the value of tasks for the purpose of reducing negative emotions, especially anxiety. Xu et al. (2018) found that emotion management and cognitive reappraisal were positively associated with online homework effort and completion amount, and with online learning satisfaction. In a study examining web-based learning (Vuorela and Nummenmaa, 2004), suppression was generally used less frequently than reappraisal. Suppression was found to be associated with an increase in dropout rates among learners of massive open online courses (Dmoshinskaia, 2016).

The use of emotion regulation during Covid-19 has been explored in studies in the fields of health psychology, media communication, and child development (Gubler et al., 2020; Rubaltelli et al., 2020; Shorer and Leibovich, 2020; Yang Y. et al., 2020). For example, Gubler et al. (2020) investigated public perception of having their lives restricted during the pandemic in Switzerland. The use of reappraisal was associated with less loneliness and higher well-being, whereas suppression was associated with higher loneliness. Reappraisal might have helped individuals to view public life restrictions in a more positive light. Conversely, suppressors might be less likely to communicate or share experiences with others and therefore felt more isolated.

**Figure 1:**
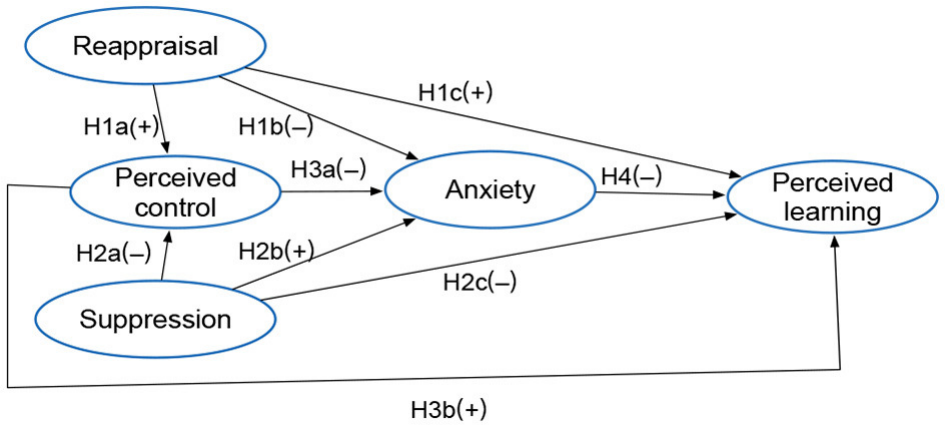
Research model and hypotheses proposed.

To the best of our knowledge, the issue of emotion regulation (cognitive reappraisal, expressive suppression), which deserves academic attention in the context of Covid-19 full remote learning, has not been explored so far. The present study sought to fill this gap by examining how reappraisal and suppression are related to remote learners' perceived control, anxiety and perceived learning. Based on the literature reviewed, we propose the following model and hypotheses (Figure 1). In addition, the present study explored the possible mediating roles of perceived control and anxiety in the association between the two types of emotion regulation and perceived learning.

**H1**: Cognitive reappraisal is positively related perceived control (H1a) and perceived learning (H1c), but negatively related to anxiety (H1b).
**H2**: Expressive suppression is negatively related to perceived control (H2a) and perceived learning (H2c), but positively related to anxiety (H2b).
**H3**: Perceived control is negatively related to anxiety (H3a), but positively related to perceived learning (H3b).
**H4**: Anxiety is negatively related to perceived learning.
**H5**: Cognitive reappraisal indirectly affects perceived learning through perceived control and anxiety.
**H6**: Expressive suppression indirectly affects perceived learning through perceived control and anxiety.

## Methods

### Context, Participants, and Procedures

On 29 January, 2020, due to the outbreak of Covid-19, the Ministry of Education of the People's Republic of China issued a policy of “suspending classes without stopping learning” (http://www.moe.gov.cn/jyb_xwfb/gzdt_gzdt/s5987/202001/t20200129_416993.html), which required students at all educational levels to take courses at home. Using a convenience sample, this study investigated a group of graduate students' perceptions of a full remote course on academic writing and research methods. They were enrolled in a medium-sized public university in southwest China. Prior to the Covid-19 outbreak, this course was routinely taught face-to-face in brick-and-mortar classrooms. During the pandemic outbreak, students took the first 13 weeks of the 18-week course (late February to late May) remotely at home, and most of them returned to university to take the course face-to-face in the 14th week.

A total of 323 graduate students enrolled in this 2-h per week course. The course was designed to improve students' academic literacy through lectures on six modules (e.g., components of an academic paper, introduction to quantitative research methods, and logic and argumentation). The course was mainly taught online (synchronously webcasted by lecturers) using Tencent Classroom (a professional webcasting platform in China's education sector), and also included synchronous and asynchronous classroom and group discussions, as well as online question and answer sessions. The course was assessed based on assignments, online class participation, and a course paper submitted at the end of the semester.

In week 13, an invitation with a link to the questionnaire was sent out via the course lecturers to invite their students to participate in this study. The introduction page of the questionnaire described the nature of this study, gave consent forms, and informed each student that his participation was completely voluntary and his response would be kept confidential. The survey was hosted on Wenjuanxing (a Chinese online platform providing functions equivalent to Qualtrics). It should be pointed out that ethics approval was not required at the time this study was conducted as per the local legislation and institutional requirements. However, informed consent was obtained from all participants.

Initially, we received a total of 272 questionnaires (response rate: 84%). Prior to the main survey, a pilot study was conducted with 15 graduate students. We removed questionnaires that were completed within 100 s based on the researchers' evaluation of the pilot study and the average time to complete the questionnaire in the main survey. In addition, we removed questionnaires completed by selecting the same option for all or the majority of the scaled measurement items, including the reversed ones. According to the above criteria, 239 valid questionnaires were entered into the analysis. Participants were between 21 and 30 years of age (*Mean* = 23.74; *SD* = 1.22), and 32% of them were male and 68% were female. They originated from different provinces and each studied at home during the time the present study was conducted. They majored in economics, business management, finance, and accounting.

### Measures

The orientation instructions of the questionnaire asked participants to indicate what they thought about and felt in relation to learning the course on academic writing and research methods during the Covid-19 remote learning period. The questionnaire consists of 32 scaled measurement items written in Chinese.

In order to minimize possible common method variance (CMV), ex-ante approaches were adopted in the design of questionnaires (Chang et al., 2010). First, as mentioned above, an introductive message was used to ensure the anonymity and confidentiality of survey takers' responses. Second, to confirm the clarity of wording, the questionnaire was pre-tested with the graduate students in the pilot study. Third, we randomized the order of items cross different constructs.

#### Emotion Regulation

We used the questionnaire developed by Gross and John (2003) to measure emotion regulation (cognitive reappraisal, expressive suppression) on a scale ranging from 1 (*strongly disagree*) to 5 (*strongly agree*). Reappraisal scale consists of six items (e.g., “When I wanted to feel less negative emotion, I changed the way I was thinking about the situation”), and suppression scale consists of four items (e.g., “I controlled my emotions by not expressing them”). The Chinese translations of these items were adopted from Chen et al. (2020).

#### Perceived Control

Students' perceived control was measured using eight items from the *Perceived Academic Control Scale* (PACS) designed by Perry et al. (2001). We slightly adapted the items to fit the present study context by changing “in my psychology course” “in my course” to “in this course” (e.g., “I had a great deal of control over my academic performance in this course”). Participants responded on a five-point Likert scale (1 = *strongly disagree* to 5 = *strongly agree*). The PACS has been used in examining online learning (e.g., Tempelaar et al., 2012; You and Kang, 2014; Buhr et al., 2019). We adopted the Chinese wording of PACS used in Ju (2012).

#### Anxiety

Anxiety was measured using eight items taken from the *Achievement Emotions Questionnaire* (AEQ) developed by Perry et al. (2005a). One sample item is *I got tense and nervous while studying this course*. Participants responded on a five-point Likert scale (1 = *not at all* to 5 = *very much*). AEQ was used to examine the emotions of online learners in previous studies (Artino, 2010; Tempelaar et al., 2012; Heckel and Ringeisen, 2019). The present study adopted the Chinese wording used in Dong and Yu (2007).

#### Perceived Learning

Perceived learning was measured using the *Cognitive, Affective and Psychomotor (CAP)Perceived Learning Scale* designed by Rovai et al. (2009). The scale was designed for both face-to-face and online learning. Given that the course was not designed to develop students' psychomotor skills, only six items measuring cognitive and affective learning were used (e.g., “I can organize course material into a logical structure”). Participants responded on a seven-point Likert scale (1 = *not at all* to 7 = *very much*).

To ensure the validity of the translation of CAP Scale, a back-translation procedure was used (Brislin, 1970). First, a professional translator who was a native Chinese speaker and fluent in English from a translation center at the university translated all original items from English to Chinese. Then, another expert translator who was also fluent in both languages translated the items back to English independently. Two of the researchers confirmed the wording of the Chinese version by comparing the two English versions.

#### Demographic Characteristics

The participants were asked to report gender and age. Gender and age were used as covariates in the present study, as evidence indicates that emotion varies with gender and age in educational contexts (Frenzel et al., 2007; Pekrun et al., 2011; Reed et al., 2014), and during Covid-19 pandemic periods (Klaiber et al., 2020; Wang et al., 2020a).

### Data Analysis Strategy

Statistical analyses were performed using SPSS v.25 and SPSS Amos v.24. We followed a two-stage approach suggested by Anderson and Gerbing (1988) to analyze the data: validating the measurement model and then the structural model. More specifically, we first assessed the reliability of each measurement scale. Confirmatory factor analysis (CFA) was then used to test the construct validity of each scale. The goodness-of-fit of measurement models were reported. After this, structural equation modeling (SEM) was conducted with gender and age as covariates to test hypotheses using the method of maximum likelihood estimation. SEM is a multivariate statistical method used to test a prior hypotheses (Kline, 2011). Model fit was estimated using the following statistical indices, as recommend by Hu and Bentler (1999): the chi-square goodness-of-fit (*x*^2^), the comparative fit index (CFI), the Tucker-Lewis index (TLI), the root mean square error of approximation (RMSEA), and the standardized root mean square residuals (SRMR). For CFI and TLI, a value >0.95/0.90 indicates a good/adequate model fit. For RMSEA, a value ≤ 0.06/0.08 indicates a good/adequate model fit, and for SRMR, a value ≤ 0.08/0.10 indicates a good/ adequate model fit.

Finally, a serial mediation analysis was performed using Preacher and Hayes (2004) method. Perceived control and anxiety served as mediators of the association between emotion regulation (reappraisal, suppression) and perceived learning. Bootstrapping with 5,000 resamples was performed to test the significance of the mediation effects. Bootstrap method was used because it does not make distributional assumptions on residuals; thus, inference can be made even if the errors do not follow a normal distribution or constant error variance (Efron and Tibshirani, 1994; Fox, 2015). All effects and bias corrected 95% confidence intervals were estimated. The effects were considered significant if the confidence interval did not contain zero. The magnitude of mediations were assessed using the ratio of the indirect effect to the total effect (Preacher and Kelley, 2011).

## Results

### Preliminary Analysis

The reliability of the five constructs were measured by Cronbach's Alpha and composite reliability. As shown in Table 1, the Cronbach's Alpha and composite reliability of each construct met the acceptable level of 0.70 (Nunnally, 1978) and 0.60 (Fornell and Larcker, 1981; Bagozzi and Yi, 1988), respectively.

**Table 1:** Factor loadings, and measurement scales reliability and validity.

**Construct**	**Cronbach's** **alpha**	**Composite** **reliability**	**Factor loadings**	**χ^**2**^**	***df***	**CFI**	**TLI**	**RMSEA**	**SRMR**
Reappraisal	0.82	0.82	0.60–0.73	14.611	9	0.986	0.977	0.051	0.030
Suppression	0.78	0.78	0.62–0.75	2.654	2	0.997	0.992	0.037	0.018
Perceived control	0.86	0.86	0.61–0.76	31.050	20	0.982	0.975	0.048	0.034
Anxiety	0.83	0.84	0.53–0.68	24.520	20	0.991	0.988	0.031	0.032
Perceived learning	0.86	0.86	0.66–0.77	22.534	9	0.975	0.958	0.079	0.033

The construct validity was measured using CFA. Standardized factor loadings and goodness of fit indexes of each construct are reported in Table 1. All indicators of each factor had loadings > 0.50 (Bagozzi and Yi, 1988). All the one-factor CFA models showed an adequate or good model fit according to the traditional cutoff criteria given above. The five-factor CFA model also showed a good model fit: χ^2^ (454) = 536.076, *p* < 0.05, CFI = 0.969, TLI = 0.966, RMSEA = 0.028, SRMR = 0.050.

Discriminant validity was checked using heterotrait-monotrait ratio of correlations (HTMT) (Henseler et al., 2015). As shown in Table 2, all HTMT values are < 0.90; thus, discriminant validity was also supported.

**Table 2:** HTMT analysis.

	**1**	**2**	**3**	**4**	**5**
1. Reappraisal	—				
2. Suppression	0.16	—			
3. Perceived control	0.48	0.31	—		
4. Anxiety	0.32	0.46	0.46	—	
5. Perceived learning	0.54	0.31	0.69	0.53	—

A single-factor test was performed to investigate possible CMV (Harman, 1976). Un-rotated confirmatory factor analysis shows that one factor explained 27% of the variance (threshold value < 50%). With caution, while CMV cannot be ruled out as a contributing factor in the present research, it did not appear to be a significant factor.

Descriptive statistics and a correlation matrix of the study variables are given in Tables 3, 4, respectively. Mean scores ranged from 3.02 to 4.86. Standard deviation scores ranged from 0.62 to 0.97. All skewness and kurtosis scores fell in the acceptable ranges of normality recommended by Kline (2011) (skewness between −3 and +3, kurtosis between −10 and +10).

**Table 3:** Descriptive statistics for main constructs.

	**Possible range**	**Mean**	***SD***	**Skewness**	**Kurtosis**
Reappraisal	1–5	3.22	0.62	−0.34	1.34
Suppression	1–5	3.02	0.67	−0.18	1.16
Perceived control	1–5	3.55	0.65	−1.00	1.35
Anxiety	1–5	3.13	0.64	−0.29	0.28
Perceived learning	1–7	4.86	0.97	−0.19	0.17

*Mean, standard deviation, skewness, and kurtosis statistics were estimated from observed variables*.

**Table 4:** Bivariate correlations for main constructs, gender, and age.

	**1**	**2**	**3**	**4**	**5**	**6**	**7**
1. Reappraisal	—						
2. Suppression	−0.13^*^	—					
3. Perceived control	0.41^**^	−0.25^**^	—				
4. Anxiety	−0.27^**^	0.37^**^	−0.39^**^	—			
5. Perceived learning	0.45^**^	−0.25^**^	0.59^**^	−0.45^**^	—		
6. Gender	−0.05	−0.14^*^	0.08	−0.05	0.04	—	
7. Age	0.08	0.10	0.05	0.06	0.01	− 0.11	—

* *p <0.05,*

** *p <0.001.*

*Gender coded as 0 = male, 1 = female*.

The results of the bivariate correlations are generally in line with our expectations. Reappraisal was positively associated with perceived control (*r* = 0.41, *p* < 0.001) and perceived learning (*r* = 0.45, *p* < 0.001), but negatively associated with anxiety (*r* = −0.27, *p* < 0.001). Suppression was negatively correlated with perceived control (*r* = −0.25, *p* < 0.001) and perceived learning (*r* = −0.25, *p* < 0.001), but positively associated with anxiety (*r* = 0.37 *p* < 0.001). In addition, perceived control was negatively correlated with anxiety (*r* = −0.39, *p* < 0.001), and anxiety was negatively associated with perceived learning (*r* = −0.45, *p* < 0.001).

### Testing the Hypothesize Model

**Figure 2:**
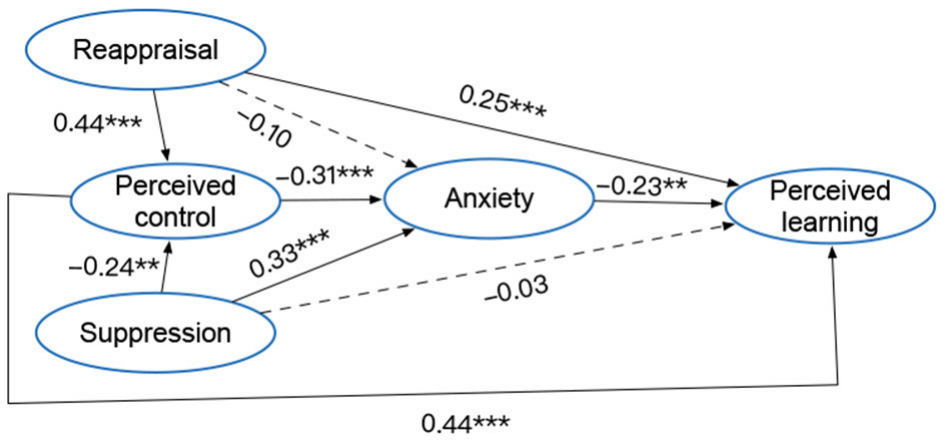
Structural model on the interplay of the study constructs. Dashed pathways are not significant; standardized coefficients are presented; **p < 0.01, ***p < 0.001. Covariates were included in the model but are not presented for simplicity.

The indices showed that the data fitted the structural model: χ^2^ (512) = 595.261, *p* < 0.05, CFI = 0.968, TLI = 0.965, RMSEA = 0.026, SRMR = 0.049. The model explained a large proportion of variance in perceived control (*R*^2^ = 0.29), anxiety (*R*^2^ = 0.32), and perceived learning (*R*^2^ = 0.56). The structural model with standardized coefficients is presented in Figure 2.

Regarding H1a and H1c, as expected, cognitive reappraisal was positively related to perceived control (H1a: β = 0.44, *p* < 0.001) and perceived learning (H1c: β = 0.25, *p* < 0.001). H1b posits that reappraisal is significantly and negatively related to anxiety. This hypothesis was not supported (*p* > 0.05). As to H2a and H2b, as anticipated, suppression was negatively related to perceived control (H2a: β = −0.24, *p* < 0.01), but positively related to anxiety (H2b: β = 0.33, *p* < 0.001). Suppression was not significantly related to perceived learning; thus, H2c was not supported (*p* > 0.05). Regarding H3a-b, perceived control was negatively related to anxiety (H3a: β = −0.31, *p* < 0.001), but positively related to perceived learning (H3b: β = 0.44, *p* < 0.001); thus, both hypotheses were supported. H4 posits that anxiety is negatively related to perceived learning, and this hypothesis was supported (H4: β = −0.23, *p* < 0.01).

### Testing Mediation Effects

Table 5 shows the mediation analysis results. We found the indirect effect of perceived control in the association between cognitive reappraisal and learning to be significant [standardized estimate (Std. estimate) = 0.197, 95% CI: 0.157, 0.382], whereas the indirect effect passing through anxiety was not (Std. estimate = 0.024, 95% CI: −0.005, 0.094). We also found a significant serial mediation effect via both perceived control and anxiety (Std. estimate = 0.032, 95% CI: 0.016, 0.094). Taken together, H5 is partially supported.

**Table 5:** Mediation analysis results.

**Route of indirect effects**	**Std.** **estimate**	**Estimate**	**Bootstrapping 95% CI**		**Ratio**
Reappraisal on learning via perceived control and anxiety
Total effect	0.500	0.631	0.441	0.832	
Direct effect **Reappraisal → Learning**	0.247	0.312	0.146	0.507	0.494
Total indirect effect	0.253	0.319	0.216	0.474	0.506
Specific indirect effect					
**Reappraisal → Control → Learning**	0.197	0.249	0.157	0.382	0.394
**Reappraisal → Anxiety → Learning**	0.024	0.030	−0.005	0.094	0.048
**Reappraisal → Control → Anxiety → Learning**	0.032	0.040	0.016	0.094	0.064
Reappraisal on anxiety via perceived control					
Total effect	−0.240	−0.186	−0.304	−0.088	
Direct effect **Reappraisal → Anxiety**	−0.103	−0.080	−0.208	0.035	0.429
Indirect effect **Reappraisal → Control → Anxiety**	−0.137	−0.106	−0.187	−0.056	0.571
Suppression on learning via perceived control and anxiety					
Total effect	−0.226	−0.330	−0.536	−0.157	
Direct effect **Suppression → Learning**	−0.026	−0.038	−0.231	0.146	0.115
Total indirect effect	−0.200	−0.292	−0.443	−0.186	0.885
Specific indirect effect					
**Suppression → Control → Learning**	−0.108	−0.157	−0.264	−0.085	0.478
**Suppression → Anxiety → Learning**	−0.075	−0.110	−0.218	−0.049	0.332
**Suppression → Control → Anxiety → Learning**	−0.017	−0.025	−0.068	−0.008	0.075
Suppression on anxiety via perceived control					
Total effect	0.400	0.358	0.218	0.518	
Direct effect **Suppression → Anxiety**	0.325	0.291	0.148	0.457	0.813
Indirect effect **Suppression → Control → Anxiety**	0.075	0.067	0.028	0.135	0.187

*Std. estimate, Standardized estimate; CI, Confidence interval; Ratio, Ratio of indirect effect (or direct effect) to total effect*.

*Confidence intervals (95% CI) that contain zero are interpreted as non-significant mediation*.

The path through perceived control alone explained 39.4% of the reappraisal-learning association, the path through anxiety alone explained 4.8% of the association, and the path through both control and anxiety explained 6.4% of the association. The results indicated that reappraisal influenced perceived learning mainly through the mediator of perceived control. Moreover, the indirect effect of perceived control in the reappraisal-anxiety association was found to be significant (Std. estimate = −0.137, 95% CI: −0.187, −0.056), and accounted for 57.1% of the association.

In the suppression-learning association, the indirect effect of perceived control alone (Std. estimate = −0.108, 95% CI: −0.264, −0.085) and that of anxiety alone (Std. estimate = −0.075, 95% CI: −0.218, −0.049) were both found to be significant. A significant serial mediation effect through both control and anxiety was also found (Std. estimate = −0.017, 95% CI: −0.068, −0.008). Thus, we found support for H6.

The percent of suppression-learning association mediated was 47.8% through perceived control alone, 33.2% through anxiety alone, and 7.5% through the path including both control and anxiety. The results suggested that suppression affected perceived learning mainly through perceived control alone and through anxiety alone. Moreover, the indirect effect of perceived control in the suppression-anxiety association was found to be significant (Std. estimate = 0.075, 95% CI: 0.028, 0.135), and explained 18.7% of the association.

## Discussion

The purpose of this study was to investigate the impact of emotion regulation strategies on control appraisal and anxiety among remote learners in the context of a global health crisis, and thus to identify the mechanisms by which anxiety arises. Students who attended the course remotely from home completed a questionnaire related to the measures of the studied variables. As expected, reappraisers perceived higher control with higher learning, whereas suppressors had lower perceived control and higher learning anxiety. Students with high perceived control experienced less anxiety and higher perceived learning. Higher anxiety was associated with lower learning. The paths of reappraisal to anxiety and suppression to perceived learning in the model were not as significant as expected. We explored the possible mediation effects of perceived control and anxiety, and all indirect effects in the association between emotion regulation and perceived learning were found to be significant, except for the indirect effect of anxiety alone in the reappraisal-learning association. According to the magnitude of mediations, perceived control was a particularly important mediator in the reappraisal-learning association, and both perceived control and anxiety were important mediators in the suppression-learning association.

A key proposition of the appraisal model (Yih et al., 2019) is that emotion regulation strategies can have an impact on appraisal of a situation (e.g., perceived control) by changing the pieces of information on which the appraisal process operates or the antecedents of the appraisal. Perceived control is one mechanism through which emotion regulation influences emotions. This proposition was supported by the existence of mediating roles of perceived control found in reappraisal-anxiety and suppression-anxiety associations in the present study. This finding is broadly consistent with that in Dijkstra and Homan (2016) study. Reappraisal and suppression are not likely to affect psychological state directly alone, but are also related to the extent of personal control over a situation, which is subsequently associated with positive or negative consequences.

As predicted by Pekrun's (2006) control-value theory, control processes are antecedents of emotions. The negative relationship between control and anxiety found in this study is consistent with previous literature (Endler et al., 2001; Pekrun et al., 2004; Butz et al., 2015). Control processes monitor emotional states, in “normal” learning situations, but also in emergency learning under a global health crisis. The importance of perceived control in the present study also lies in its direct effect on perceived learning and in its mediating role in the association between emotion regulation and learning. Individuals with high perceived control hold an attitude of responsibility for results (Rotter, 1966). They believe that learning outcomes largely depend on the effort they put in and actively respond to the challenges posed by changes. During the pandemic, high control learners might have actively taken various methods to solve the problems encountered in full remote learning, such as strict time management and removal of distractions from learning (Gelles et al., 2020). These measures are likely to have facilitated perceived learning.

Consistent with previous literature (Jamieson et al., 2010, 2018), reappraisers perceived more learning in the present study. Reappraisers are more likely to hold a *stress-is-enhancing* mindset, flexibly viewing stress as an opportunity for growth (Hagger et al., 2020). According to *broadening and building* theory (Fredrickson, 1998), the positive mindset held by reappraisers helps to broaden thought–action repertoire. Adapting learning to a specific, as-yet-unencountered environment requires cognitive flexibility, and a positive mindset is beneficial to the development of this flexibility. In addition, our participants who used more reappraisals had higher perceived control and then had lower anxiety, which may explain the observed serial mediational pathway associating reappraisal with learning through both perceived control and anxiety.

Chronic attempts to avoid an unwanted thought can lead to an increase in the frequency, severity, and accessibility of that thought (Wegner et al., 1987; Lavy and Van den Hout, 1990). This paradoxical effect of suppression elucidates a positive relationship between suppression and anxiety. Suppression is also detrimental to interpersonal relationships. Suppression decreases the likelihood of emotional sharing, social support, and relationship closeness (Gross, 2002; Gross and John, 2003). Studies of loneliness in individuals during Covid-19 showed that suppressors were more likely to feel lonely (Gubler et al., 2020). Loneliness might be one of the factors that contributed to increased anxiety during the pandemic (Banerjee and Rai, 2020). In addition, some Covid-19 remote students felt anxious due to the fear of falling behind their peers (Pakpour et al., 2020). Suppression of emotions prevented adequate communication between remote learners to understand each other's learning progress, in which case they perceived themselves as learning less effectively than others.

Similar to Low et al.'s (2017) study 2 in general psychology, the authors found that spontaneous use of emotional suppression did not directly predict success, but indirectly influenced success via a negative emotion (depression). Our participants' use of expressive suppression did not directly and significantly lead to a decrease in perceived learning. More use of suppression was associated with higher anxiety, which was then associated with less perceived learning. Our data provide preliminary evidence showing an important mediating role of anxiety in the relationship between suppression and perceived learning in educational contexts. Additionally, we found support for the existence of a serial mediation linking suppression to learning through both perceived control and anxiety.

According to the *attentional control theory* (Eysenck et al., 2007), high anxiety affects the inhibitory function responsible for suppressing irrelevant information and reduces the attention given to the task being performed. For high-anxiety learners, attention is focused on anxious reactions rather than on processing learning tasks. Anxiety experienced during Covid-19 full remote learning was shown to be negatively related to perceived learning. This result is consistent with some studies (Artino et al., 2010; You and Kang, 2014), but not with others (Tempelaar et al., 2012; Heckel and Ringeisen, 2019). Our participants reported a higher anxiety mean (3.13) than the theoretical median (3, middle value of a five-point Likert scale). The participants, overall, had more than moderate levels of anxiety. Additionally, some studies have clearly shown exacerbated anxiety during Covid-19 (Husky et al., 2020; Wang et al., 2020a,b; Arribathi et al., 2021). One explanation for the negative relationship between anxiety and perceived learning found in the current study may be related to our participants' anxiety levels.

This study has several limitations that we would like to highlight. First, the results of a study based on a convenience sample may limit its generalizability or external validity. Our study was conducted on a full remote course taken by graduate students at a university in China during Covid-19. Because of the differences in the specific implementation of emergency remote teaching across countries and grade levels (Hall et al., 2020), it remains to be examined whether the results of this study can be replicated in other countries or at other educational levels. The second limitation is that the present study used learners' self-reported data. Concerns about the reliability and validity of studies using this type of data have been discussed in the literature (e.g., Demetriou et al., 2015), including over- and under-reporting and social desirability bias (Gonyea, 2005). Future research could consider also using more objective data, such as logs and test scores, to provide a more comprehensive examination of learners' behaviors, emotional experiences, and academic performance. Third, although the current study used existing instruments to measure reappraisal and suppression, these instruments were not developed specifically for emergency learning situations or even academic contexts, which may reduce the validity of the measurement instruments used. There is a need to confirm the current results in future studies using questionnaire instruments developed specifically for the investigation of emergency learning contexts.

Despite these limitations, this study provides some insight into teaching and learning during emergencies. These findings highlight the need for interventions to reduce remote learners' anxiety. Training in emotion regulation has been shown to be effective in improving the ability to cope with various emotions and reducing anxiety (De Witte et al., 2017). It is thus recommended that researchers, classroom teachers, mental health providers, and school authorities design and develop (preferably Internet-mediated) training programs aimed at developing the adoption of healthy emotion regulation strategies in students with low levels of adaptive regulation. Moreover, perceived control was found to be an important antecedent of anxiety and perceived learning. Educators can adopt instructional strategies aimed at enhancing students' perceived control over full remote learning. For example, they can provide technical support to students when needed, give emotional support to ensure that students are actively engaged in learning, and teach students how to properly use social media for communication through training sessions (Sobaih et al., 2020).

Finally, the implications of this study for future research are as follows. According to the process model of emotion regulation (Gross, 1998, 2002; Gross and Thompson, 2007), regulatory strategies consist of five families. We focused only on reappraisal and suppression. Situation selection, attentional deployment, and situation modification were not explored in this study, so it would be worthwhile to investigate how these strategies affect perceived control and achievement emotions in ERL. Moreover, the relationship between anxiety and perceived learning is not consistent across studies (e.g., Artino et al., 2010; Tempelaar et al., 2012; You and Kang, 2014; Heckel and Ringeisen, 2019). We speculate that their relationship is moderated by learning contexts. It would be useful to examine which constituents of learning contexts (e.g., type of course, course difficulty, assessment method) moderate the relations between anxiety and perceived learning.

## Conclusion

This study examined the effects of two emotion regulation strategies on perceived control over learning and anxiety in a sample of graduate students taking a remote course during the pandemic. The results showed that cognitive reappraisal was positively related to perceived control and perceived learning. Expressive suppression was negatively related to perceived control, but positively related to anxiety. Anxiety was significantly, negatively related to perceived learning. We found the existence of different patterns of mediation in the pathways from the two types of emotion regulation to perceived learning. These results were interpreted in the light of the model that aims to unify emotion regulation and perceived control, the control-value theory and the attentional control theory. For learners in emergency remote learning situations, rather than holding emotions inside, understanding and accepting emotions and allowing them to be regulated using cognitive reappraisal can promote control over a situation, healthy emotional development, and learning outcomes.

## Data Availability Statement

The raw data supporting the conclusions of this article will be made available by the authors, without undue reservation.

## Ethics Statement

Ethical review and approval was not required for the study on human participants in accordance with the local legislation and institutional requirements. The patients/participants provided their written informed consent to participate in this study.

## Author Contributions

TZ: conceptualization, methodology, data analysis, and writing. ZF and XL: conceptualization, writing, and editing. LY: data analysis, writing, and editing. WH: methodology. All authors contributed to the article and approved the submitted version.

## Conflict of Interest

The authors declare that the research was conducted in the absence of any commercial or financial relationships that could be construed as a potential conflict of interest.
